# Whole-body muscle MRI in patients with spinal muscular atrophy

**DOI:** 10.1007/s00415-025-13005-3

**Published:** 2025-03-16

**Authors:** Sophia Vera Frølich, Noémie Receveur, Nanna Scharff Poulsen, Adam Espe Hansen, John Vissing

**Affiliations:** 1 Copenhagen Neuromuscular Center, Department of Neurology, Rigshospitalet, Blegdamsvej 9, 2100 Copenhagen, Denmark; 2 Department of Radiology, Rigshospitalet, Copenhagen, Denmark

**Keywords:** Spinal muscular atrophy, Bulbar function, Magnetic resonance imaging, Fat replacement, Tongue fat fraction

## Abstract

**Background:**

Spinal muscular atrophy (SMA) is a motor neuron disease with loss of musculature, which is replaced by fat. Previous magnetic resonance imaging (MRI) studies have focused on imaging muscles either in lower or upper extremities, but whole-body MRI can provide additional information on the involvement pattern. This study examined whole-body muscle fat replacement and the relationship between muscle structure, function, and bulbar symptoms.

**Method:**

We conducted a descriptive, cross-sectional study. We assessed the fat replacement in skeletal muscles using whole-body MRI, the muscle function using the Motor Function Measurement 32, and bulbar muscle strength using the Bulbar Rating Scale. The presence of bulbar symptoms and function was assessed using the Voice Handicap Index, Eating Assessment Tool questionnaires, and a swallowing test.

**Results:**

We recruited 20 adult patients with type II and III SMA. The most affected muscles were the psoas major, soleus and rectus femoris, while the least affected muscles were the biceps brachii, deltoideus, and pterygoideus medialis. The tongue was involved in nearly half of the patients. Most patients reported issues with swallowing (75%) and voice (95%) but had relatively preserved strength of bulbar muscles.

**Conclusion:**

Certain muscles are more prone to fat replacement than others in SMA, with a predominant proximal–distal and extensor-flexor involvement. Nearly half of the patients had increased fat content in the tongue, which is associated with dysphagia. In addition, most patients retained muscle strength in the bulbar muscles, despite advanced muscle weakness in the rest of the body.

## Introduction

Spinal muscular atrophy (SMA) is a progressive, neurodegenerative disease caused by pathogenic variants in the survival motor neuron 1 (*SMN1*) gene on chromosome 5. Insufficient SMN1 protein expression leads to lower motor neuron degeneration, resulting in extensive muscle atrophy and muscle weakness and replacement of muscle mass by fat. Proximal muscles are typically more affected than distal muscles, and upper extremities are more spared than lower ones in early stages [1–4]. As the disease progresses, some experience respiratory muscle weakness [5, 6], leading to assisted ventilation, and weakness of the bulbar muscles, causing swallowing difficulties and nutritional support [7]. These complications increase morbidity and mortality due to aspiration pneumonia [7, 8], restrictive lung disease [6], and malnutrition [8].

The bulbar symptoms are well-described in patients with type I SMA, but less so in type II and type III, even though these symptoms also affect them. It is described that SMA type II and III can experience reduced maximal mouth opening [9] and chewing, swallowing [7, 8, 10, 11], and voice impairment [12] due to a combination of muscle weakness of mouth-opening muscles, abnormalities in craniofacial morphology, and reduced range of motion in the jaw [10, 13, 14]. Despite this, only one study has investigated the bulbar muscles with magnetic resonance imaging (MRI) [9], and none have investigated the tongue.

Whole-body MRI is increasingly used in neuromuscular disorders, but previous MRI studies in SMA have mainly focused on muscle involvement in either the upper [15, 16] or lower extremities and mainly in segments [1, 2, 4, 17–26]. Only a few studies have considered both parts [3, 27] and none have conducted whole-body muscle MRI on a cohort. Whole-body MRI can provide additional information on other body segments and enhance the understanding of SMA-specific involvement patterns. This information has recently become more important since therapies have become available for some patients with SMA. The therapies increase life expectancy, especially when administered in the early stages of the disease [28–31]. Treatment efficacy varies, possibly due to differences in muscle involvement, with a better response in persons with preserved muscles [28–31]. Thus, the decision to prescribe drug treatment is based on muscle function and the possibility of preserving it. However, if patients with severe muscle affection still have preserved bulbar function, they can potentially benefit from treatment. If we can preserve bulbar function, it might extend both lifespan and quality of life. Therefore, it is essential to consider the overall muscle function alongside bulbar symptoms and define the extent and pattern of muscle involvement in patients with SMA [2].

The aim of this study is to conduct whole-body MRI in ambulant and non-ambulant patients with type II and III SMA, and to characterize potential patterns of muscle involvement.

## Methods

### Participants

The patients were recruited from the Copenhagen Neuromuscular Center, Rigshospitalet, Denmark, and through The Danish Rehabilitation Center for Neuromuscular Diseases (RCFM). Inclusion criteria were genetically verified diagnosis of SMA type II or III and age over 15 years. Exclusion criteria were competing disorders that could interfere with the results, such as other muscular disorders, and exclusion from MRI if MRI was contraindicated.

### Study design

We performed a descriptive cross-sectional study between January 2024 and August 2024.

The primary outcome was the whole-body intramuscular fat content using MRI. Secondary outcomes were correlations between muscle structure and muscle function, bulbar function, and lung function.

### Magnetic resonance imaging

We performed a whole-body MRI scan, using a 3.0 T MAGNETOM Vida system “Syngo MR XA50” (Siemens Healthineers). The MRI protocol included a localizer, T1-weighted, and 2-point Dixon sequences, generated in the transverse and sagittal planes. Breath-hold was used for Dixon sequences of the thorax and abdomen. Subjects were placed in a head-first supine position with coils covering their body and head. Coils were individually placed due to the patients’ altered body composition, which included severe scoliosis and contractures. We used positioning pads to ensure that the patients were comfortable during the scans. Contractures in some patients prevented the acquisition of high-resolution images of the muscles as positioning the body parts correctly for scanning was challenging.

The following protocol settings were used:

*T1 head, thorax, abdomen, pelvis, leg* (Field of view (FOV) 550 mm; slice thickness 6 mm; slice gap 2%; echo time (TE) repetition time (TR) 10,000/333,000 ms).

*Dixon head* (FOV 330–521 mm; slice thickness 210–366 mm; slice gap 0.2%; TE/TR 1230/3970 ms).

*Dixon thorax* (FOV 330–521 mm; slice thickness 186–288 mm; slice gap 0.2%; TE/TR 1230/3970 ms).

*Dixon abdomen* (FOV 330–521 mm; slice thickness 186–312 mm; slice gap 0.2%; TE/TR 1230/3970 ms).

*Dixon pelvis* (FOV 330–521 mm; slice thickness 108–312 mm; slice gap 0.2%; TE/TR 1230/3970 ms).

*Dixon leg* (FOV 330–521 mm; slice thickness 258–372 mm; slice gap 0.2%; TE/TR 1230/3970 ms).

### Skeletal muscles analysis

Images were assessed using the Horos software v.3.3.6. Investigators (SVF and NR) scored the fat replacement on T1-weighted images using the Mercuri score and outlined the muscles on Dixon images. Afterward, a third investigator (NSP) corrected the scorings and outlining to increase precision and ensure uniformity; disagreements were solved by consensus.

**Fig. 1:**
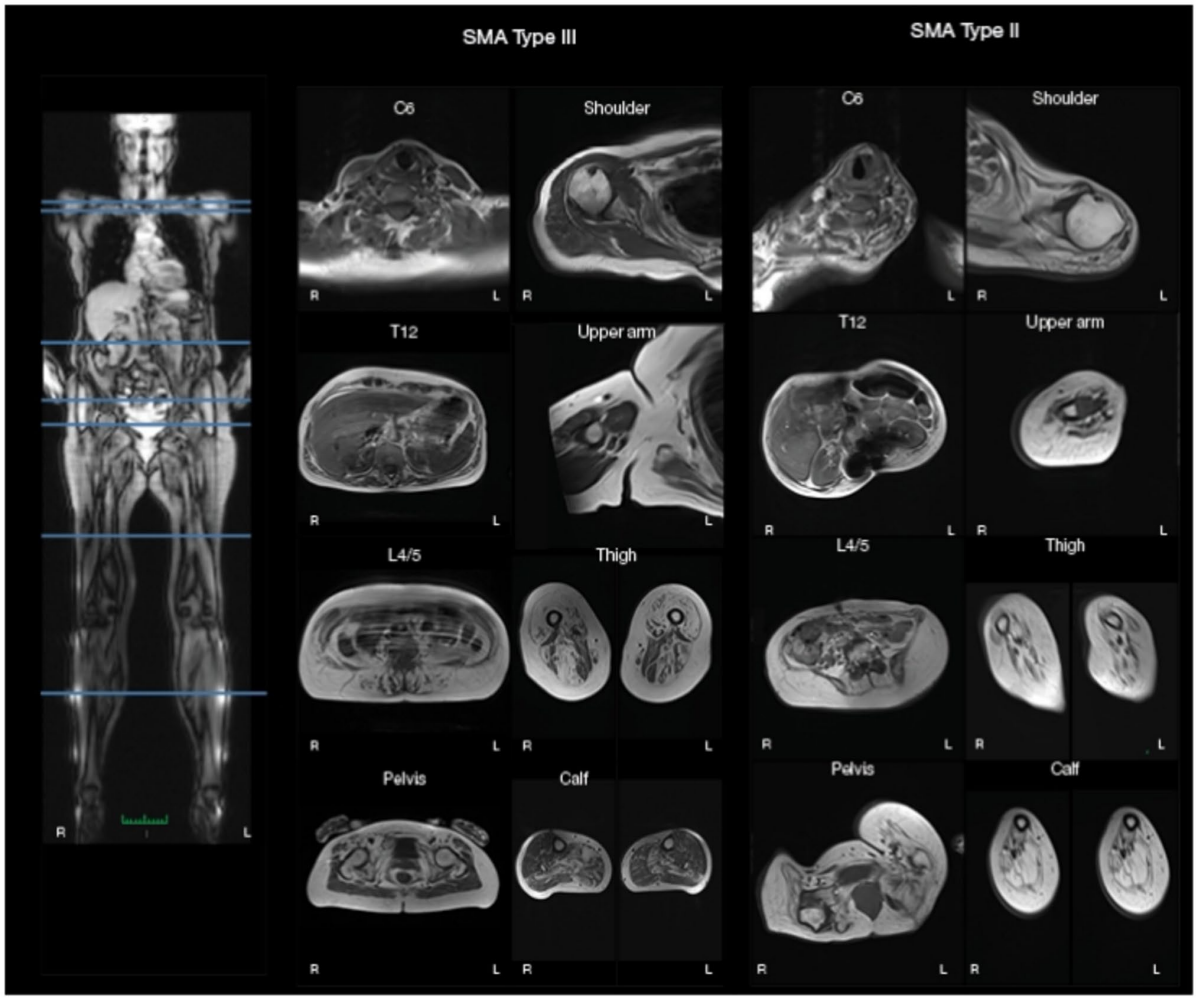
Region of interest of muscles using whole-body MRI. Magnetic resonance imaging T1-weighted and Dixon sequences were analyzed at each region of interest as illustrated with the lines on the whole-body scan to the left. In the middle is shown T1-weighted sequences of the muscles at the region of interests for a patient with SMA type III and to the right a patient with SMA type II

**Fig. 2:**
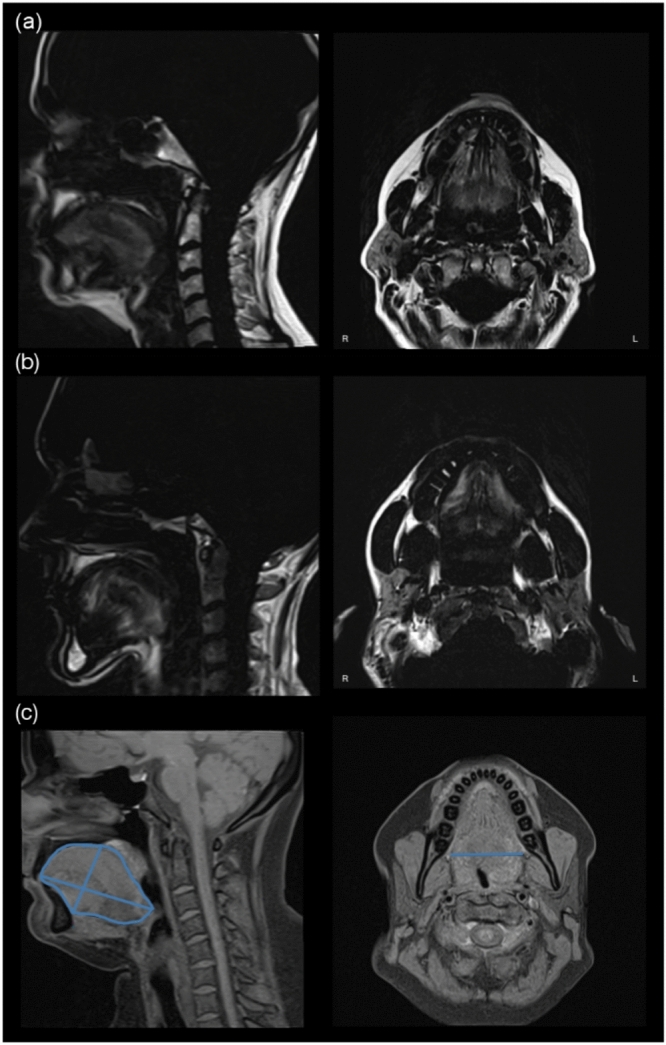
Magnetic resonance imaging of the tongue. MRI of the tongue in sagittal and transverse planes in a patient with SMA type 3 (**a**) and a patient with SMA type 2 (**b**) in a Dixon in-phase sequence. The tongue showing axial, anterior–posterior, midsagittal diameters, and midsagittal area (**c**) in a T1-weighted sequence

The following sections were scored in the transverse plane: the shoulder at level with the top of the humeral head, the upper arm at the widest part of the muscles, the spine at C6, T12 and L4/L5, the pelvis at level with the top of the femoral head, the thigh at 50% of the length of the femur, and the calf at 33% of the tibias length proximally (Fig. 1). In addition, the muscles of the face were assessed at level with the tongue and nasal cavity, while the infrahyoid muscles were assessed at the C7 level of the spine. The tongue was assessed both in the midline of the sagittal plane and in the transverse plane (Fig. 2).

The following muscles were evaluated: *head* (infrahyoid, tongue, and mastication muscles including masseter and pterygoideus lateralis et medialis), *Axial* (trapezius, intrinsic cervical muscles, transversospinalis, erector spinae, psoas major), shoulder (deltoideus, infraspinatus, subscapularis), *upper arm* (biceps brachii, brachialis, triceps brachii), *pelvis* (gluteus maximus), *thigh* (rectus femoris, vastus medialis, vastus intermedius, vastus lateralis, sartorius, gracilis, adductor longus, adductor magnus, semitendinosus, semimembranosus, biceps femoris), and *calf* (tibialis anterior, extensor hallucis longus, extensor digitorum longus, fibularis brevis et longus, gastrocnemius lateralis et medialis, soleus, posterior compartment (tibialis posterior, flexor digitorum longus, flexor hallucis longus)) (Fig. 1).

#### Analysis of T1-weighted images

The intramuscular fat replacement was scored from the T1-weighted images using the Mercuri score [2]:

- Stage 0: normal muscle.
- Stage 1: small areas of increased density.
- Stage 2a: increased density in less than 30% of the total muscle volume.
- Stage 2b: increased density in 30–60% of the total muscle volume.
- Stage 3: washed-out appearance.
- Stage 4: end-stage appearance.

#### Analysis of Dixon images

The Dixon images were used to calculate the fat fraction: fat fraction (FF) = (signal fat/signal in) × 100, except in the tongue where it was calculated as FF = signal fat/(signal fat + signal water) × 100. The individual muscles were outlined whenever possible, as in some cases, the fat fraction was too high to distinguish between different muscles making outlining impossible. If the muscle was not visible in the region of interest (Fig. 1), it was outlined in either a higher or lower level. It was not possible to outline the individual infrahyoid muscles, and therefore, their fat fraction was not calculated.

#### Tongue dimensions

In the sagittal plane, the anteroposterior diameter was measured as the longest distance from the apex to the base of the tongue. The midsagittal diameter was measured as the longest distance from the surface to the genioglossus muscle and the midsagittal area was determined in the midline. The axial diameter of the tongue was measured as the longest diameter in the axial direction on the transverse plane (Fig. 2). Reference values from a former study were used, in which 36 controls were MRI scanned. The anteroposterior diameter was 7.127 $\pm$ 0.66 cm and the midsagittal area was 25.23 $\pm$ 3.69 ${\text{cm}}^{2}$ [32].

### Muscle function

The muscle function was assessed with the Motor Function Measurement 32 (MFM32). Each of 32 items were scored on a scale from 0 to 3, where 0 = cannot initiate the task, 1 = partially performs the task, 2 = performs the movement incompletely, or completely but imperfectly, and 3 = performs the task fully and normally. The score is divided into three domains: (1) standing and transfers, (2) axial and proximal motor function, and (3) distal motor function. The total score ranges from 0 to 100%, with higher scores meaning higher level of function. The test was performed by three certified raters NSP, NRC, or SVF.

### Bulbar function

We used the Bulbar Rating Scale (BRS) to evaluate bulbar muscle strength [33]. The scale scores from 8 (worst) to 32 (normal). Due to contracture of the jaw, it was difficult to assess “soft palate elevation” and “posterior pharyngeal wall constrictor”. During the test, the rater also looked for fasciculations of the tongue. The test was performed by NSP, NRC, or SVF.

Swallowing function was assessed by measuring the time needed to swallow 80 ml of water at 5 °C. Duration > 7 s was considered abnormal [33].

Voice and swallowing evaluations were conducted using the validated self-reported questionnaires Voice Handicap Index (VHI) and Eating Assessment Tool 10 (EAT-10). The patients completed the questionnaires independently, without any member of the study group present. Those unable to mark the questionnaires themselves received assistance from their caregiver. The VHI consists of 30 items divided into 3 subscales: functional (10 items), physical (10 items), and emotional (10 items). Responses are rated on a five-point scale (0 = never, 1 = almost never, 2 = sometimes, 3 = almost always, 4 = always). The total score ranges from 0 to 120, with scores of 0–30 indicating mild impairment, 31–60 moderate impairment, and 61–120 severe impairment [34]. EAT-10 is a questionnaire consisting of 10 items, using a five-point scale ranging from 0 = no issue to 4 = severe issue. The total score ranges from 0–40, with scores greater than 3 indicating issues with swallowing efficiency and safety.

### Lung function

The lung function was investigated using a spirometer (Vitalograph micro spirometer Model 6300).

Test results included forced vital capacity (FVC), forced expiratory volume (FEV), and their predicted percentage based on age, weight, and height. In cases where the patients were unable to hold the spirometer themselves, either their caregiver or a member of the study team assisted with holding it. The subjects who were unable to close their teeth over the mouthpiece only closed their lips around it.

### Clinical assessment

We documented the medical history of all patients including SMA type, sex, age, weight, height, respiratory and/or nutritional support, medication for SMA, and history of scoliosis surgery.

### Statistical analyses

The study is a descriptive study, which is why a power calculation was not performed. Since MRI was used to assess our primary outcome and almost all patients with SMA have an increased amount of intramuscular fat, we tried to recruit as many adult patients as possible from SMA type II and III to cover the range of phenotypical variation.

Statistical tests were conducted using R Studio v. 2200.12.0. The data distribution was evaluated using normal probability plots. Depending on the distribution, continuous variables are represented as mean or median, whereas categorical data are expressed as counts or percentages. Categorical variables from the questionnaires were visualized using violin plots with box plots. Correlations are presented using Spearman’s correlation coefficients. Missing values were handled by case-wise deletion.

Artwork was created using Adobe InDesign, Illustrator, and PowerPoint.

## Results

### Participants

Forty-four patients were invited to participate in the study. Seventeen did not respond, two did not want to participate, five met the exclusion criteria, and one patient was not tested due to practical reasons. Two patients contacted us through RCFM, leaving twenty-one included participants. One patient dropped out of the study.

Of the remaining 20 patients, 11 had type II and 9 had type III SMA. The patients ranged in age from 22 to 72 years (median: 32 years, interquartile range (IQR): 11 [25–36]). Seven were women and thirteen were men (Table 1). However, three of the included patients did not undergo MRI due to the following reasons: severe back pain when lying down on the MRI table, metal that could not be confirmed as MR compatible, and a piercing that could not be removed. Six patients were undergoing treatment with Risdiplam (Evrysdi), with a mean treatment duration of 205 days.

**Table 1:** Patient characteristics

Patient number	SMA type	Sex	Age	BMI	S/NS/W	MFM32	Respiratory support	Nutritional support	Risdiplam	Scoliosis surgery
1	II	M	22	14.6	NS	35.4	1	0	Yes	Yes
2	II	M	24	18.4	S	29.2	1	1	Yes	Yes
3	II	M	36	27.5	NS	22.9	1	1	No	Yes
4	II	M	22	19.1	NS	32.3	1	1	Yes	Yes
5	II	M	23	12.7	NS	18.8	1	2	Yes	Yes
6	II	M	30	21.7	S	10.7	2	2	No	Yes
7	II	M	36	13.8	NS	20.8	0	1	No	Yes
8	II	M	57	20.4	NS	0.0	1	1	No	Yes
9	II	M	33	20.9	NS	37.5	1	0	No	Yes
10	II	M	26	23.9	NS	26.0	0	1	Yes	Yes
11	II	M	25	11.1	NS	25.0	2	2	No	Yes
12	III	F	59	37.6	S	50.0	1	0	No	No
13	III	F	42	29.3	S	45.8	0	0	No	Yes
14	III	M	34	24.6	S	60.4	0	0	No	No
15	III	F	32	28.5	S	37.5	0	1	No	Yes
16	III	F	26	33.3	W	86.5	0	0	No	No
17	III	F	33	33.1	W	80.2	0	0	No	No
18	III	F	32	39.5	S	54.2	0	1	No	No
19	III	M	25	17.7	S	36.5	1	1	Yes	Yes
20	III	F	72	24.3	W	81.2	0	0	No	No

*BMI* body mass index, *F* female, *M* male, *NS* non-sitters, *S* sitters, *W* walkers

Nutritional support: 0 = none, 1 = need assistance while eating, such as having food cut into small bites or avoiding certain types of food, 2 = percutaneous endoscopic gastrostomy (PEG) tube. Respiratory support; 0 = none, 1 = noninvasive, 2 = invasive

### Magnetic resonance imaging

#### Muscle structure of the whole body

**Fig. 3:**
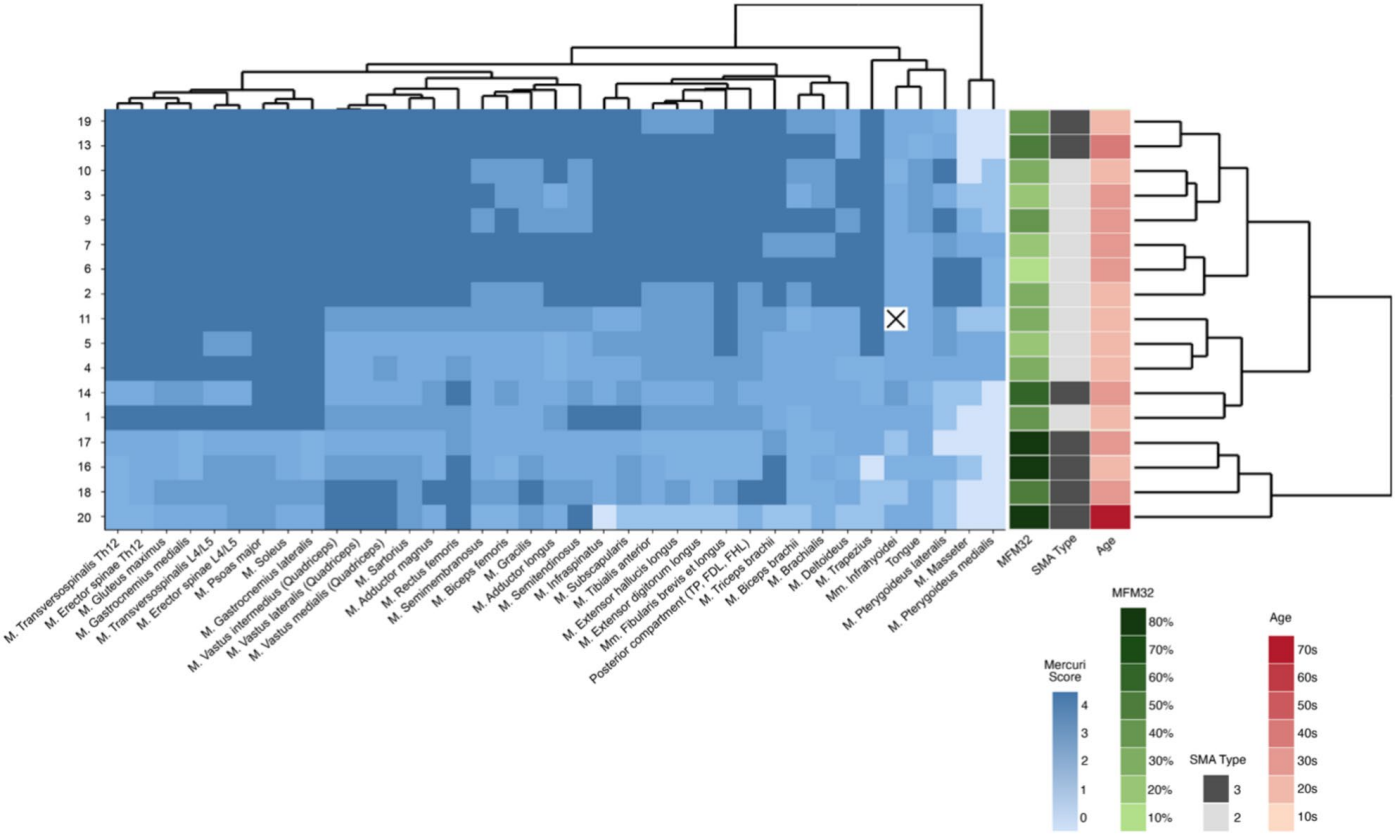
Heatmap of intramuscular fat replacement of whole body and tongue. Mercuri score on T1-weighted images of the muscles including the tongue conducted with whole-body MRI in patients with spinal muscular atrophy Type II and III. The shading of the colors indicates the severity of involvement of the muscles, with light colors indicating milder involvement and darker colors indicating more severe involvement. The white box with black cross indicates missing data for patient no. 11, as the infrahyoid muscles could not be assessed due to tracheostomy. *MFM32* motor function measurement 32, *SMA* spinal muscular atrophy type II or III

**Fig. 4:**
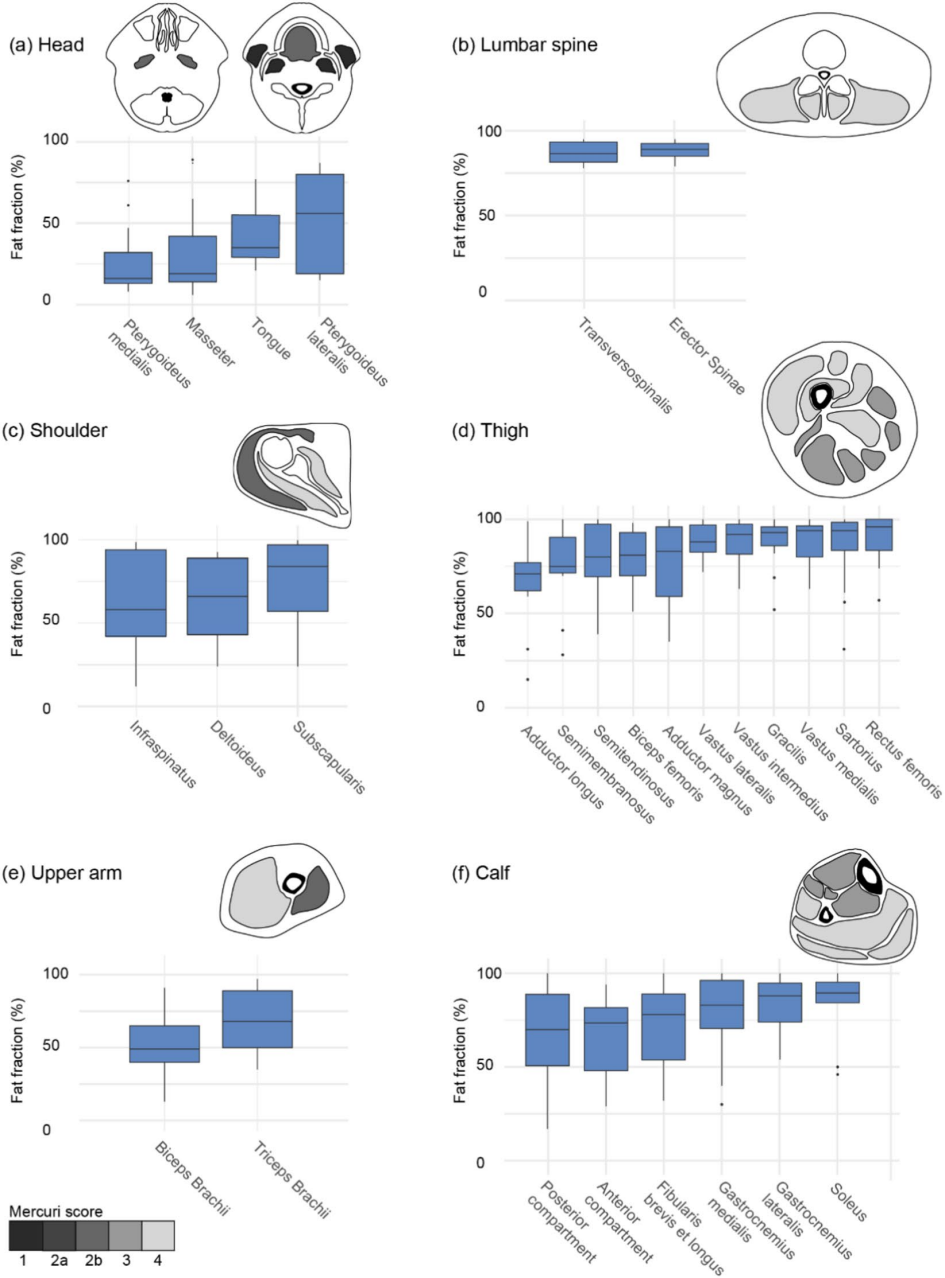
Detailed overview of the whole-body muscle involvement pattern in (**a**) head, (**b**) lumbar spine, (**c**) shoulder, (**d**) thigh, (**e**) upper arm, and (**f**) calf. Illustrations show fat replacement scored using the Mercuri score (median values) on T1-weighted images, where the level of fat replacement is illustrated using gray colors. Boxplots show muscle fat fraction calculated from outlined Dixon images. The illustrations contain data from MR scans of 17 patients. The number of patients included in the boxplots varies due to difficulties outlining end-stage muscles: head *n* = 17, shoulder *n* = 13, upper arm *n* = 13, lumbar spine *n* = 8, thigh *n* = 11, and calf *n* = 10

Using the Mercuri score on the T1-weighted images, we found that the most affected muscles overall were the psoas major, soleus, and rectus femoris while the most spared muscles were the biceps brachii, deltoideus, and pterygoideus medialis (Figs. 3 and 4). Patients with high fat content generally had type II SMA and a low MFM32 score, with no relation to age (Fig. 3).

- In the *head*, the pterygoideus medialis was the least affected muscle, followed by the masseter and the infrahyoid muscles. Pterygoideus lateralis was the most affected muscle in all patients.
- The *tongue* had a Mercuri score equal to or higher than 2a in all patients.
- In the *spine*, the intrinsic cervical muscles were more spared than the thoracic and lumbar muscles (erector spinae, transversospinalis).
- In the *shoulder*, the deltoideus was spared, whereas subscapularis and infraspinatus were more affected.
- In the *upper arm*, the biceps brachii was more spared than the brachialis and triceps brachii.
- In the *pelvis*, the gluteus maximus was affected in all patients.
- In the *thigh*, the most affected muscles were the anterior compartment (rectus femoris, quadriceps vastus lateralis et medialis et intermedius), except for sartorius which was relatively spared. The medial muscles were less affected (adductor magnus et longus, gracilis). The most spared muscles were the semimembranosus and biceps femoris.
- In the *calf*, were the anterior compartment (tibialis anterior, extensor hallucis longus, and extensor digitorum longus) less affected than the rest of the calf. The gastrocnemius and soleus were the most affected muscles, followed by the deep posterior compartment (tibialis posterior, flexor digitorum longus, flexor hallucis longus). Meanwhile, the lateral muscles (fibularis longus et brevis) were moderately affected (Figs. 3 and 4).

Dixon analysis was performed to quantify the fat replacement of the individual muscles to better distinguish the involvement pattern between muscles of identical Mercuri score (visualized in Fig. 4). The pattern of muscle involvement observed with Dixon analysis (median fat fraction values) was generally consistent with that observed using the Mercuri score, with some minor differences: the most prominent differences were that the pterygoideus lateralis seemed less severely affected than the tongue with Dixon, conversely on T1, and that sartorius and gracilis seemed more affected with Dixon than with T1. Based on the Mercuri score, the deltoideus appears to be less affected than the infraspinatus, and the anterior compartment of the calf less affected than the deep posterior compartment, though the differences are minor. In the box plots (Fig. 4) based on the Dixon method, there is such a large overlap between the fat in the muscles that they can likely be considered equally affected.

#### Tongue size

The results are summarized in Table 2. Three patients exhibited a high anteroposterior diameter (7.931 cm, 7.962 cm, and 7.801 cm). In addition, one of these patients, along with another, showed a high midsagittal area (29.468 ${\text{cm}}^{2}$ and 29.371 ${\text{cm}}^{2}$).

**Table 2:** Tongue size

	SMA type II (*n* = 10)	SMA type III (*n* = 7)
Anteroposterior diameter (cm)	6.676 $\pm$ 0.497	6.979 $\pm$ 0.867
Midsagittal diameter (cm)	5.348 $\pm$ 0.586	5.519 $\pm$ 1.126
Axial diameter (cm)	5.052 $\pm$ 0.438	4.749 $\pm$ 0.430
Area midsagittal (cm)	23.512 $\pm$ 2.469	25.956 $\pm$ 3.248

MRI parameters of the tongue shown as mean $\pm$ sd values. Reference values from a former study were used, in which 36 controls were MRI scanned. The anteroposterior diameter was 7.127 $\pm$ 0.66 cm and the midsagittal area was 25.23 $\pm$ 3.69 cm^2^ [32]

### Muscle function

At time of investigation, 45% were non-sitters, 40% were sitters, and 15% were walkers. The total score of the MFM32 ranged from 0% to 81.2% (median: 35.9, IQR: 26.6 [24.5–51.0]). The domain with the lowest scores was standing and transfers (median: 0, IQR: 2.6 [0–2.6]), then axial and proximal motor function (median: 45.8, IQR: 50.7 [27.1–77.8]), while the domain with the highest scores was distal motor function (median: 80.95, IQR: 34.5 [60.7–95.2]).

### Bulbar function

#### Bulbar rating scale

**Fig. 5:**
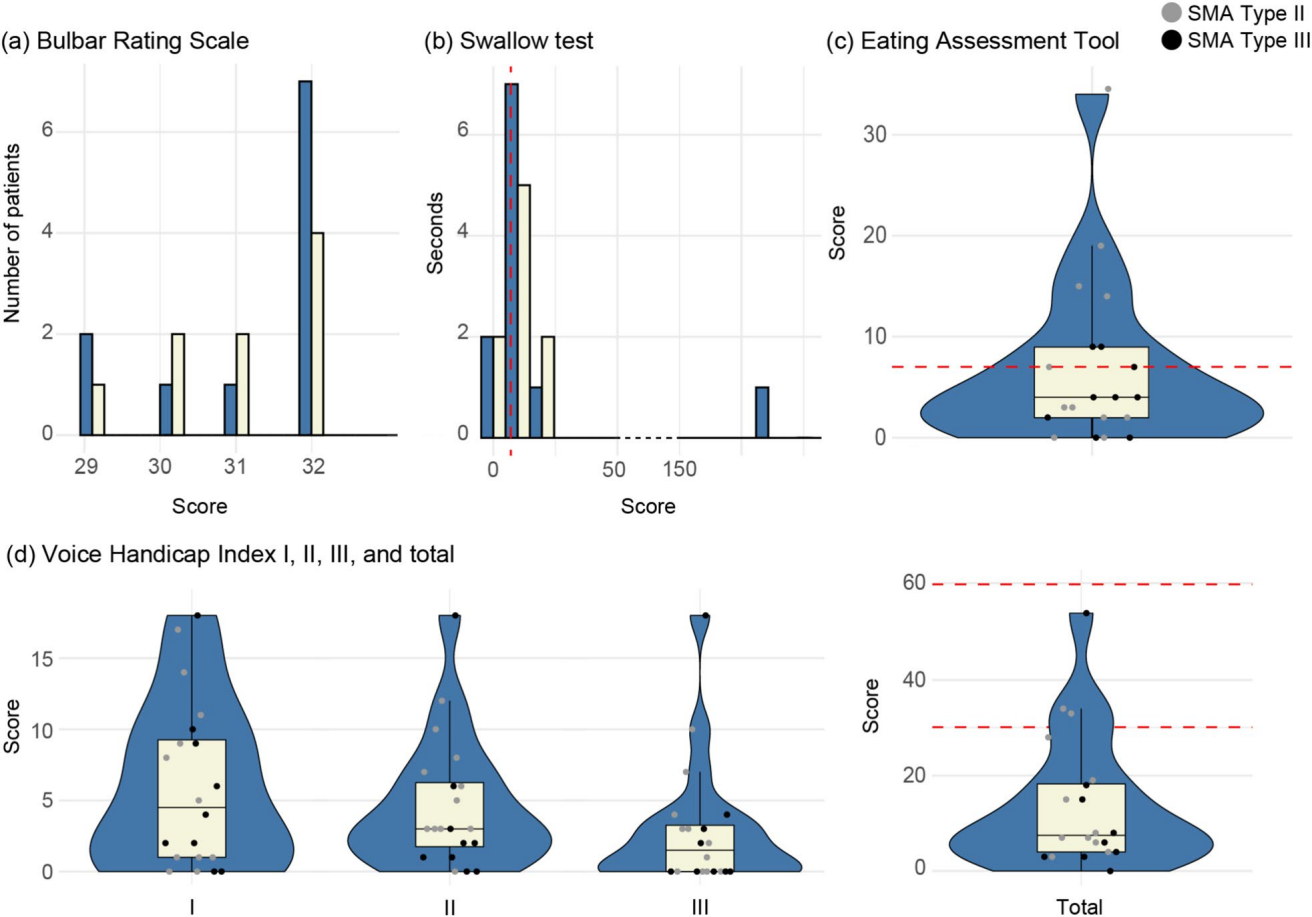
Bulbar function in patients with spinal muscular atrophy. Figure showing the results from questionnaires on bulbar function, including swallowing, eating, and voice-related problems. **a** swallow test, **b** eating assessment tool 10, **c** bulbar rating scale, **d** voice handicap index, *VHI-I* = functional dimension, *VHI-II* = physical dimension, *VHI-III* emotional dimension, *VHI-total* total score. The data points illustrate the distribution of the patients’ scores. The dotted line in **a** marks a 7-s score, below which is considered normal. The dotted line in **b** marks a 3-point score, below which is considered normal. The dotted lines of **d** total mark scores of 30 points and 60 points, which are the thresholds between mild, moderate, and severe voice impairment

All patients achieved high scores on the BRS, ranging from 29 to 32 (median: 32, IQR: 2 [30–32]), indicating preserved muscle strength in the bulbar muscles (Fig. 5a). Bulbar weakness (BRS score < 32) was observed in eight (42%) patients. Problems due to reduction of tongue strength were found in five patients (26.3%), affection of orbicularis oris in five patients (25%), reduction of jaw opening in four patients (21%) defined as unable to open ≤ 2 stacked fingers, reduction of tongue protrusion in two patients (10.5%), and affection of orbicularis oculi in one patient (5.3%). During examination, fasciculations of the tongue were observed in 12 patients (60%).

#### Swallow test

Ten patients (50%) had a swallowing time exceeding 7 s, which is the upper limit for normal duration (Fig. 5b). The duration varied widely, ranging from 3 to 189 s, with a median of 7.5 s (IQR: 6 [5.75–11.75]). The patient with a high swallowing time (patient no. 5 in Table 1) required pauses during the test, while others managed to swallow all the water in one attempt. Two patients, who required invasive respiratory support (patients’ no. 6 and 11 in Table 1), were able to breathe while drinking, which potentially could bias a quicker swallowing function.

#### Eating assessment tools

Fifteen (75%) patients reported problems with swallowing on EAT-10, and out of these, twelve (60%) patients scored greater than or equal to 3, indicating problems with swallowing efficiently and safely (Fig. 5c). The most frequently reported problems included:

- When I swallow food sticks in my throat (65%).
- Swallowing solids takes extra effort (55%).
- Swallowing pills takes extra effort (55%).
- I cough when I eat (45%).

During clinical evaluation, all patients were asked about difficulties with speech, swallowing, and chewing. Twelve patients self-reported experiencing dysphagia, and an additional twelve patients avoided specific types of food due to swallowing issues. Eleven patients reported chewing problems, while seven patients reported problems with speaking.

#### Voice handicap index

Nineteen (95%) patients scored mild-moderate impairment on the Voice Handicap Index; out of these, seventeen (85%) patients scored on the functional dimension (VHI-I), eighteen (90%) on the physical dimension (VHI-II), and twelve (60%) on the emotional dimension (VHI-III) (Fig. 5d). The most frequently reported problems were:

- People have difficulty understanding me in a noisy room (75%).
- My voice makes it difficult for people to hear me (70%).
- I run out of air when I talk (65%).
- The sound of my voice varies throughout the day (60%).
- People ask me to repeat myself when speaking face-to-face (55%).
- My family has difficulty hearing me when I call them throughout the house (50%).

Sixteen patients (80%) scored between 0 and 30 indicating mild impairment, three patients (15%) scored between 31 and 60 indicating moderate impairment, and none scored between 61 and 120 indicating severe impairment [34].

### Lung function

We performed lung function tests on 19 patients. Data for one patient is missing due to an oversight on the test day. Fifteen (75%) patients had a low predicted FEV1 percentage and twelve patients (60%) had a low predicted FVC percentage (both outcomes below the normal values of 80% [35]). Among these, 12 patients had both FVC and FEV below 50%, while 2 patients had both below 20%, indicating a very low lung function.

### Correlations between muscle structure and muscle and bulbar function

**Fig. 6:**
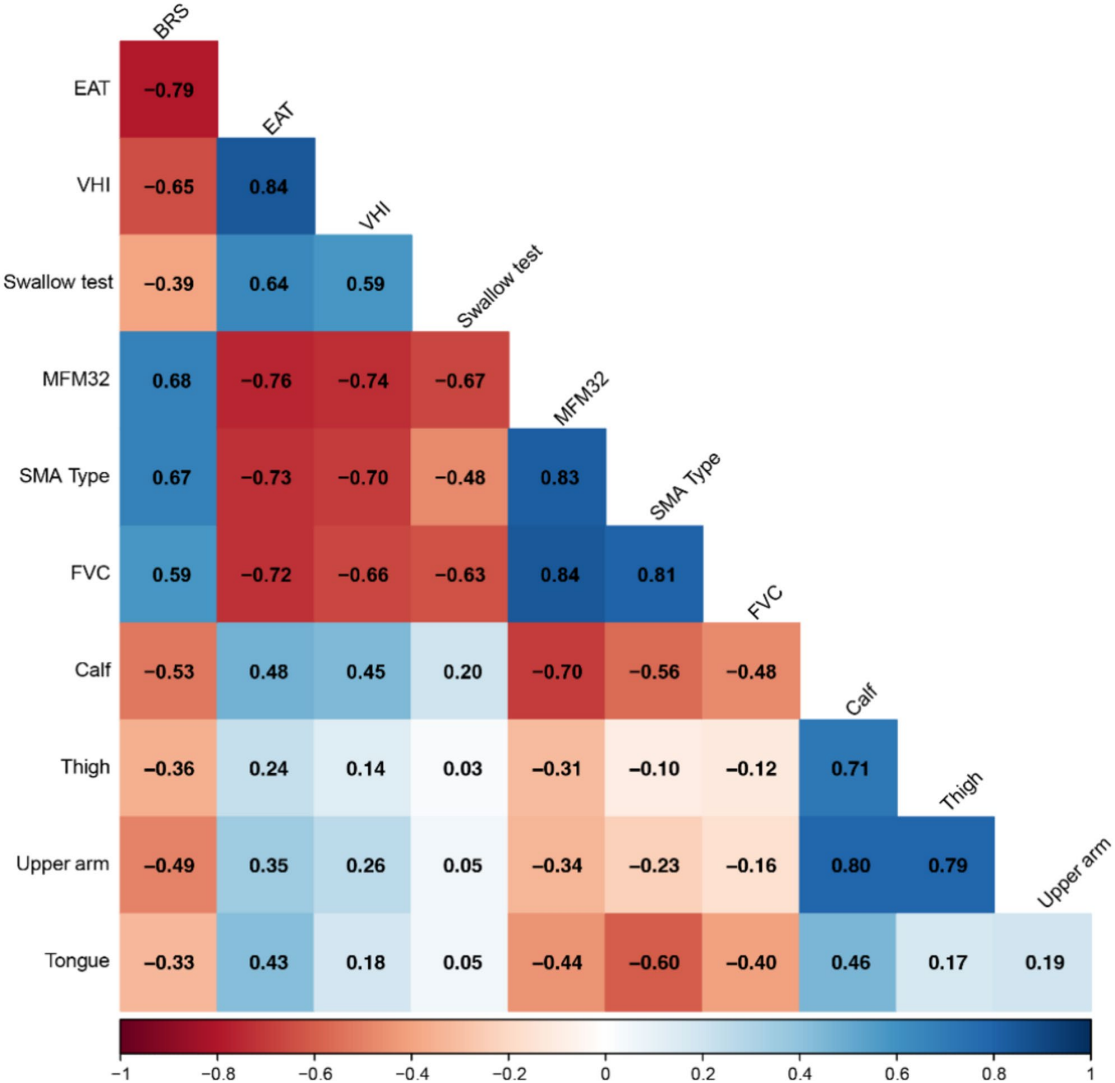
Correlations between muscle structure and muscle and bulbar function. Correlogram of Spearman correlations between muscle structure (using results from Mercuri score on T1-weighted MRI images) and muscle and bulbar function. The numbers represent the correlation coefficients. The dark colors indicate strong positive and negative correlations. A correlation coefficient > 0.7 is considered strong, between 0.4 and 0.7 moderate, and < 0.4 low correlations. *BRS* bulbar rating scale, *EAT* eating assessment tool 10, *FVC* forced vital capacity, *MFM32* motor function measurement 32, *SMA* spinal muscular atrophy type II or III, *VHI* voice handicap index. Calf, thigh, upper arm, and tongue represent the mean fat content in the muscles, using Mercuri score

The correlogram (Fig. 6) shows a strong correlation between the fat content in the muscle groups of the upper arm, thigh, and calf. The fat content of the tongue shows a moderate correlation with the fat content of the calf. In addition, the fat content in the calf and tongue correlate negatively and moderately with the severity of the disease (estimated from MFM32, SMA type, and lung function (FVC)). This correlation is low for the fat content in the thigh and upper arm. Furthermore, the fat content in the tongue is moderately correlated with dysphagia (EAT), while low correlation was observed with voice impairment (VHI). The fat content in the calf and upper arm is moderately correlated with bulbar muscle strength (BRS).

There is a moderate correlation between the bulbar muscle strength (BRS) and the overall muscle function (MFM32). These are both moderately (BRS) or strongly (MFM32) correlated to SMA type, indicating that higher muscle function is associated with higher SMA type (III).

The bulbar muscle strength shows a strong to moderate correlation with voice impairment (VHI) and dysphagia (EAT) indicating that preserved muscle strength in the bulbar muscles is associated with fewer bulbar symptoms. Furthermore, there is a moderate correlation between the swallow test and dysphagia. Overall, there is a correlation between dysphagia and voice impairment, as well as bulbar muscle strength with the severity of the disease: low MFM32 score, lung function, and SMA type (II).

## Discussion

This study contributes to a comprehensive phenotyping of patients with spinal muscular atrophy types II and III by investigating fat replacement of skeletal muscles throughout the entire body, including the tongue, and correlating these findings to the bulbar function and lung function.

### Proximal–distal and extensor-flexor muscle involvement

Fat replacement of muscles is common in most neuromuscular disorders and is often specific to the disease [36]. SMA is a disease with predominant proximal involvement and in more severe cases diffuse involvement. The pattern of muscle involvement has previously been described in SMA patients for specific body segments [1–4, 37], but for the first time, we have examined muscles across the entire body, revealing fat replacement patterns in skeletal muscles, which enhance the understanding of SMA-specific involvement patterns.

Overall, we found that the mastication muscles (medial and lateral pterygoideus, masseter) and the upper arm muscles were less affected compared to those in the thigh, calf, and back. We observed that at the thigh level, the extensors (quadriceps and rectus femoris) were more affected, while the adductor muscles and flexors (biceps femoris, semimembranosus, and semitendinosus) were more spared. The gracilis and sartorius appeared more spared using the Mercuri score compared to Dixon analysis. This discrepancy might be due to errors in muscle outlining for these smaller, peripheral muscles. Despite this difference, the muscles are relatively affected in both cases. In addition, the difference between the two methods could be due to the smaller number of patients included in the Dixon analysis compared to the Mercuri scoring. In the calf, the soleus and gastrocnemius were consistently affected, indicating that the extensors were more affected than the flexors. A similar pattern was seen in the arm, where the triceps brachii was always more affected than the biceps brachii. The spine muscles were more severely affected at the thoracic and lumbar levels compared to the cervical level, which was relatively spared. The gluteal muscles in the pelvis were always affected.

The pattern of fat replacement is more distinct in patients with preserved muscle mass, as with disease progression, all muscles are eventually replaced by fat. The sparing of individual muscles observed is not related to the *SMN1* gene variant, as a similar pattern has been reported in other genetic forms of the disease [2, 38], but rather due to differences in motor neuron involvement [2]. One patient showed more distal than proximal involvement even though he has a homozygote deletion in exon 7 and exon 8 in the *SMN1* gene. Predominant distal involvement is observed in other genetic forms of SMA but has been reported in only a few patients with the *SMN1* mutation [2, 39].

Selective neuronal vulnerability in SMA refers to the phenomenon, that specific motor neurons and the corresponding muscles they innervate, are affected in a distinct pattern. This selective involvement has been demonstrated in the present and other MRI studies [1–4, 15–28]. The underlying mechanisms are unknown, yet understanding them is important, as treatment efficacy depends on both treatment duration and the vulnerability of the motor neurons [40]. Preclinical studies in mouse models have shown variability in loss of neuromuscular junctions, contributing to differential vulnerability [40–43]. Other studies highlight variations at the spinal cord level [43, 44]. In addition, factors such as motor unit size [43], lack of axonal sprouting [42, 45], and whether the muscles are fast-synapsing or delayed-synapsing [40, 41] have been proposed as contributing factors.

A study [40] reported higher levels of neuromuscular junction loss in the muscles from the core, neck, and proximal limbs, while distal limbs and head muscles where less affected. Similarly, another study [43] found increased vulnerability of motor neurons innervating proximal and axial muscles. These findings are consistent with our observations of fat replacement pattern, where proximal and axial muscles are more affected, and masticatory and distal muscles remain relatively spared.

### The fat content in the calf is the best indicator of disease severity

We found that overall, the fat content in the calf most strongly correlates with muscle function, lung function, and SMA type, indicating that the fat content in the calf is the best indicator of disease severity. This may be because, in most patients, the thigh muscles were almost entirely replaced by fat, while the calf muscles showed more variability in fat content, allowing for detectable correlation. We did not find the same correlation with the fat content in the thigh and upper arm. This underlines the predominantly proximal muscle involvement resulting in the thigh and upper arm muscles being more severely affected at an earlier stage of the disease. Age did not show an association with muscle involvement, suggesting that while SMA progresses over time, age-related progression is not the most dominant factor for the level of muscle involvement.

We found that the fat content in the calf correlates with fat content in the tongue, bulbar muscle strength, and symptoms such as voice and swallowing difficulties, as well as reduced lung function. The fat content in the calf serves not only as the best indicator of disease severity, but also as indicator for the overall function, including lung function and bulbar function.

### The bulbar function correlates with the muscle function of the extremities

The mechanism underlying bulbar impairment involves multiple factors impacting voice, swallowing, chewing, and lung function. Previous studies have shown that some patients have decreased maximal mouth opening due to fat replacement in the mouth-opening muscles [8, 9]. In addition, muscle contractures in the temporomandibular joint limiting jaw motion [10] and abnormal cranial morphology, characterized by protruding maxilla and a receding mandible [10] affect bulbar function, potentially leading to malocclusion and nutritional deficiencies.

We found that most patients experienced problems with their voice (94.7%) and swallowing (78.9%) and that patients with voice impairment often also had swallowing difficulties. This is correlated with the strength of the bulbar muscles as well as the overall muscle function of the extremities. However, most patients had preserved strength in the bulbar muscles assessed by BRS despite severely affected muscle function elsewhere. This may be due to that, in addition to the bulbar muscles, the pharyngeal and laryngeal muscles also play a role in voice and swallowing functions.

Our findings on bulbar symptoms are consistent with previous studies reporting high rates of bulbar impairment [7–11, 14]. One study found that 94% of patients reported voice impairment on VHI, and 85% reported dysphagia on EAT-10. This together with the most frequently reported items on the questionnaires aligns closely with our findings. In our study, one of the most reported issues (55%) was difficulty swallowing solids rather than liquids (10%). This finding has also been reported in earlier studies [7, 11, 12]. Patients with SMA have muscle weakness, and swallowing solids requires more muscular strength than swallowing liquids. This differs from dysphagia in patients with upper motor neuron lesions, where difficulty swallowing liquids is more pronounced [11].

We studied both subjective and objective measures of oro-bulbar function. For voice, most participants (95%) reported subjective perception of voice difficulties, which aligned with the objective, structural findings of increased fat content of muscles involved in voice production (the tongue and infrahyoid muscles). For swallowing, most participants (75%) reported swallowing problems, again aligning with the increased fat content of muscles involved in swallowing (the tongue, infrahyoid and masticatory muscles). However, only half of the participants showed reduced bulbar muscle strength and prolonged swallowing time. MRI of the tongue, infrahyoid, and masticatory muscles showed selective involvement, with pterygoideus lateralis and the tongue being severely affected, while the infrahyoid, pterygoideus medialis, and masseter were relatively preserved. The complexity of swallowing, involving multiple muscles and coordination, may explain these discrepancies between subjective and objective measures of function.

Interestingly, a recent study in children and adults with SMA [46] found that fewer patients reported subjective difficulties with swallowing and mastication than what was observed by objective findings. This contrasts with our results, where subjective were more pronounced than the objective findings. This discrepancy may be due to differences in age or methods used to assess symptoms and objective findings and emphasize the importance of addressing both subjective and objective oro-bulbar function in the clinical setting.

### The increased muscle fat content in the tongue is moderately associated with dysphagia

We found increased fat content in the tongue in nearly half of the patients using MRI. The average fat content in the tongue is normally higher in healthy individuals than in other muscles. One study [32] found that the normal fat content in the tongue is 33% (SD $\pm$ 4.0%); this is higher than reported in other, smaller studies [47, 48]. Considering this average and standard deviation, eight (47%) patients had an abnormally high fat content.

The fat content in the tongue correlated with SMA type and motor function as well as high fat content in the calf, suggesting that higher fat content in the tongue is seen in patients with more severe disease stages. We did not find correlations between the fat content in the tongue and voice impairment or swallow duration. However, we did find a moderate correlation between the fat content in the tongue and dysphagia (Fig. 6). This could be due to other factors contributing to swallowing function, such as the pharyngeal muscles, in addition to the tongue.

We found that only four patients had a larger tongue compared to controls from another study [32]. It is important to note that height is a substantial factor in tongue size [32]. Since patients with SMA are shorter than the control group due to severe scoliosis, we may have underestimated the frequency of enlarged tongues.

BMI is commonly used as an indicator of nutritional status and metabolic health, but its reliability can be questioned, particularly in patients with SMA. Altered body composition, including severe scoliosis and wheelchair dependency, often lead to underestimation of height, while the progression of the disease results in significant loss of muscle mass, contributing to low body weight. Conversely, in milder forms like type III, where muscle loss is less pronounced, overweight is more common, when expressed by BMI. In addition, regardless of weight, many patients lead a sedentary lifestyle due to muscle weakness. Altogether BMI may not reflect their metabolic health.

Despite its limitations, BMI remains an easily accessible marker and is, therefore, commonly used in clinical practice. Over half (54.5%) of the patients with type II SMA in our study required assistance with eating, such as cutting food into small pieces or avoiding certain types of food, and three patients relied on a PEG tube. In comparison, one-third (33.3%) of patients with type III SMA needed eating assistance, and none had a PEG tube. Underweight (BMI < 18.5 ${\text{m}}^{2}$) was more common in type II SMA (45%), with only one patient being overweight (BMI > 25 ${\text{m}}^{2}$), whereas 54.5% of patients with type III SMA were overweight, with just one being underweight. Notably, underweight patients were more likely to require eating assistance, reflecting their lower muscle mass.

### The lung function is often reduced and associated with muscle function and structure

Most of the patients had reduced lung function, consistent with previous findings [5, 6]. We found that this reduction is associated with bulbar symptoms and bulbar muscle strength. The association between lung function and bulbar symptoms indicates that patients with bulbar symptoms are at higher risk of respiratory complications. In addition, reduced lung function correlates with the disease severity (MFM32 and SMA type), as the progressive weakening of the respiratory muscles results in reduced respiratory capacity. Initially, the intercostal musculature is primarily affected, followed by the diaphragm [10]. This can result in an inward paradoxical collapse of the rib cage, as the intercostal muscles become unable to stabilize the ribcage against the diaphragm’s force during inspiration [10]. Over time, this may lead to a bell-shaped chest, which combined with scoliosis, further restricts chest expansion and impairs lung function [10].

### Strength and limitations

Limitations of the study include the small number of patients. The inclusion of a larger patient group would have provided more valid results but would have required international collaboration since SMA is rare. Men were overrepresented in the SMA type II group, where all ten patients were male, whereas women dominated in the SMA type III group (Table 1). This small number of patients may introduce bias, as our study population may not be representative of the overall SMA population.

We did not include cough peak flow measurements, as we assessed lung function solely through spirometry, using FEV1 and FVC. This method does not account for secretion management, which is an issue in oro-bulbar function and an indicator of the risk for respiratory complications.

We did not perform muscle function tests or MRI scans of healthy controls, which may influence the comparative value and interpretations of these values. Other limitations include that the muscle visualization on MRI was suboptimal due to contractures and severe scoliosis resulting in less ideal patient positioning during the scans. All type II patients had undergone scoliosis surgery, which made scoring the back muscles challenging due to artifacts.

A strength of this study is that we did not exclude patients due to contractures or limited motor functions as other MRI studies have done before; this gives a better representation of the disease spectrum as more severe phenotypes are included.

### Future perspectives

The findings of fat replacement patterns in skeletal muscles observed in our group suggest the need for further investigation in a larger group of SMA patients over time to better understand disease progression. This is particularly important as the phenotype will evolve with new treatment possibilities. Emphasis should be placed on enhancing our understanding of the mechanisms behind this muscle involvement pattern.

## Conclusion

In conclusion, the use of whole-body MRI provided us with information about the SMA-specific fat replacement pattern in the skeletal muscles, showing extensor-flexor and proximal–distal patterns of involvement. The fat content in the calf is the best indicator of disease severity and overall function including lung function and bulbar functions. The results confirm a selective vulnerability in SMA, with a complex pattern of muscle involvement. This is seen in the masticatory muscles, where the lateral pterygoid is severely affected, while the medial pterygoid and masseter is relatively preserved, despite extensive muscle impairment in the rest of the body.

We found that almost all patients reported subjective difficulties with speaking and swallowing, whereas only around half of the patients had objectively reduced strength in the bulbar muscles and a prolonged swallowing time. Although all patients had advanced stage fat replacement of lower extremity muscles, there was considerable variability in the involvement of the oro-bulbar muscles. Most patients exhibited only a mild increase in intramuscular fat, while a few demonstrated severe accumulation. These findings suggest that the extremity muscles are affected earlier than the oro-bulbar muscles, which undergo fat replacement at later stages. This emphasizes the need to consider and preserve bulbar function, even in patients with severe muscle impairment in the extremities. If we can preserve bulbar functions, it can potentially extend both lifespan and quality of life for patients with SMA.

## Data Availability

Data can be shared upon reasonable request to the corresponding author.

## Abbreviations

BMI: Body mass index
BRS: Bulbar rating scale
EAT-10: Eating assessment tool 10
FEV1: Forced expiratory volume
FF: Fat fraction
FVC: Forced vital capacity
IQR: Interquartile range
MFM32: Motor function measurement 32
MRI: Magnetic resonance imaging
NS: Non-sitters
RCFM: The Danish rehabilitation center for neuromuscular diseases
S: Sitters
SMA: Spinal muscular atrophy
SMN1: Survival motor neuron 1
W: Walkers
